# Aspirin in the prevention of preeclampsia: A protocol for systematic review and meta analysis

**DOI:** 10.1097/MD.0000000000027916

**Published:** 2021-12-03

**Authors:** P.Z. Mkhize, W.N. Phoswa, O.P. Khaliq, V. Dorsamy, J. Moodley

**Affiliations:** a Department of Obstetrics and Gynecology, School of Clinical Medicine, College of Health Sciences, Nelson R Mandela School of Medicine, University of KwaZulu-Natal, Durban, South Africa; b Department of Life and Consumer Sciences, University of South Africa (UNISA), Science Campus, Florida, Roodepoort, South Africa; c School of Laboratory Medicine and Medical Sciences College of Health Sciences, Nelson R Mandela School of Medicine, University of KwaZulu-Natal, Durban, South Africa.

**Keywords:** aspirin, compliance, low-dose, maternal and fetal outcome, preeclampsia, pregnancy, prevention

## Abstract

**Introduction:**

Aspirin is widely used to prevent pregnancy related vascular disorders such as preeclampsia (PE), intrauterine growth restriction and maternal disorders. However, the indications for the use of aspirin during pregnancy is currently controversial because the dosage of aspirin used and the sample sizes in various studies differ considerably. Furthermore, women of African ancestry are more likely to have higher rates of PE and more severe cases than those of their Caucasian counterparts. Yet, there are very few studies in this population group. Therefore, the aim of this review will be to determine the effect of low-dose aspirin (LDA) for prevention of PE in women of African ancestry.

**Methods and Analysis:**

This is a protocol for a systematic review and meta-analysis of published studies on the effect of LDA for prevention of PE. Relevant information will be accessed from the following databases; PubMed, Cochrane Central Register of Controlled Trials, Google Scholar, Google, EBSCO Host, and the Web of Science. The studies will be mapped in 2 stages: stage 1 will map studies descriptively by focus and method; stage 2 will involve additional inclusion criteria, quality assessment and data extraction undertaken by 2 reviewers in parallel. Evidence will be synthesized using relevant systematic research tools. Meta-analysis and subgroup analysis will be conducted using RevMan whilst Stata 13 will be used for meta-regressions. We will follow recommendations described in the preferred reporting items for systematic reviews and meta-analyses statement and the Cochrane Handbook for Intervention Reviews.

**Discussion:**

The use of LDA as a prophylactic treatment has been considered for the prevention of PE. However, studies evaluating the use of LDA in women of African ancestry are few. Therefore, with the increase in the prevalence of PE in the African population, it is critical to further investigate the use of LDA in pregnant women of African ancestry.

**Ethics and dissemination:**

The review and meta-analysis will not require ethical approval and the findings will be published in peer-reviewed journals and presented at local and international conferences. The findings of this review will inform all stakeholders on current and future guidelines on the use of aspirin in pregnancy, especially in populations of African ancestry.

**Systematic review registration:**

International prospective Register of Systematic Reviews (PROSERO) number: (CRD42020213213).

## 1 Introduction

Hypertensive disorders of pregnancy (HDP) such as preeclampsia (PE) and eclampsia are major contributors to maternal and perinatal morbidity and mortality worldwide.^[1]^ PE is a multisystem disorder of pregnancy typically characterized by a systolic blood pressure of 140 mm Hg or more and a diastolic blood pressure of 90 mm Hg or more taken on 2 separate measurements at least 4 to 6 hours apart with/without proteinuria diagnosed after 20 weeks of gestation.^[2]^ PE is also accompanied by other features of maternal organ dysfunction such as acute kidney injury, liver dysfunction, neurological deficit and haematological disturbances, or utero-placental dysfunction.^[2]^ PE is currently classified as a 2-stage disorder with the first stage involving abnormal placentation occurring early in the first trimester followed by “maternal syndrome” in the second and third trimesters as evidenced by a change in the levels of antiangiogenic factors, namely elevated levels of soluble FLT and a decrease in placental growth factor, resulting in an antiangiogenic milieu.^[3]^

Aspirin is a cyclooxygenase inhibitor that has anti-inflammatory properties. It is commonly used as prophylaxis against the development of or delay in the onset of PE in women that are at risk.^[4]^ Treatment with aspirin in high-risk normotensive pregnant women reduces the frequency of PE by 3%.^[5]^ Randomized controlled trials have shown that low-dose of aspirin (LDA) reduced the risk of PE substantially.^[6]^ There still exists uncertainty around the timing of initiation of the drug and effective dose, and this may differ between population groups and geographic locations.

The dosage of aspirin used influences the risk of PE. A study by Meher et al^[7]^ reported a reduction in risk of PE of 10%, while another reported a reduction by 50% (100 mg per day at 16 weeks gestation). A larger multi-center trial confirmed that 150 mg aspirin per day from 11 to 14 up to 36 weeks gestational age showed >60% reduction.^[8]^ However, a study published in 2018 reported that a daily dose of aspirin (100 mg) may have different effects on the risk of placental abruption if the onset of treatment begins prior to 16 weeks gestation rather than ≥16 weeks.^[9]^

Even though LDA is promulgated for safe use in women at risk of PE, the timing and duration of use is important. The American College of Obstetricians and Gynecologists and the Society for Maternal–Fetal Medicine recommends prophylactic low dose aspirin commencement after 12 weeks^[10]^ In low- and middle-income countries, lack of access to healthcare, inadequate education and poor socio-economic status are associated with the development of PE.^[11]^ These factors are also responsible for later initial visits to antenatal facilities thereby reducing the protective effect LDA may have had if commenced as recommended. Furthermore, reduced antenatal care visits and lack of insight into its use may reduce compliance and adherence to the therapy.

In addition, a study conducted in women of African ancestry reported that hypertension is associated with salt sensitivity and a reduction in nitric oxide-dependent vasodilation. A decrease in nitric oxide levels were commonly found in African Americans than other race groups.^[12]^

Women of African ancestry are prone to HDP. Apart from the socioeconomic and equitable healthcare disparity between indigenous Africans and other populations, there may be infectious and genetic predispositions to PE.^[12]^ It is therefore important that studies assessing at risk reduction strategies to combat PE focus on population specific factors. Clinical trials evaluating LDA use conducted in women of African ancestry are few. Systematic reviews and meta-analyses conducted recently have pooled studies from global LDA studies reports which may overshadow the effect of studies conducted in African region.^[8,10,13]^ It is therefore important to conduct more LDA studies focusing on women of African ancestry.

Moreover, aspirin compliance has a vital role in the detection of its effectiveness. It is believed that true suboptimal response to aspirin is notable from aspirin noncompliance. A systematic review and meta-analysis conducted in 2018 revealed that LDA administration at ≤16 weeks of gestation given to women at risk of PE was allied with a reduction in maternal and neonatal adverse outcomes, compared to placebo or no treatment. Low dose aspirin therapy was also associated with an improvement in fetal growth.^[13]^ Other systematic reviews report that if aspirin is prescribed universally without screening, it would probably largely reduce overall compliance rates resulting in the weakening of compliance in high risk women.^[14]^ It is believed that compliance testing should be based on detection and quantification of aspirin metabolites from maternal blood or urine.^[15]^

## 2 Research questions

a. Has there been sufficient numbers of women of African ancestry included in studies using LDA for the prevention of PE, and have these studies included techniques or evaluated compliance and adherence?
b. Is there a reduction of adverse outcomes in pregnant women consuming LDA between 14 and 36-weeks’ gestation?

## 3 Objectives

a. To evaluate LDA as a treatment for primary prevention of PE in all pregnant women considered to be at high risk following the first-antenatal visit.
b. To evaluate the effects of LDA on the incidence of early (delivery before 34 weeks of gestation) PE, the incidence of intrauterine growth restriction, fetal death, perinatal death, and placental abruption

## 4 Methods

This study will be a systematic review with meta-analysis of published articles. The protocol will follow recommendations described in the preferred reporting items for systematic reviews and meta-analyses^[16]^ statement and the Cochrane Handbook for Intervention Reviews.^[17]^ Statement and article screening and selection process will also be demonstrated through a preferred reporting items for systematic reviews and meta-analyses-protocol flow diagram. Furthermore, the current protocol has been registered with the International Prospective Register of Systematic Reviews (PROSPERO): CRD42020213213.

## 5 Eligibility

### 5.1 Study design

This systematic review and meta-analysis will include matched cohort, cohort, case control, observational and randomized control trial with a clearly defined population and interventions used. While, observational studies, reviews, case studies, and animal studies will be excluded in this study.

### 5.2 Participants

Pregnant women of African Ancestry.

### 5.3 Intervention

Aspirin.

### 5.4 Comparator

Pregnant women on Placebo.

### 5.5 Outcomes

Gestational hypertension, PE, eclampsia, HELLP syndrome, fetal growth restriction, placenta abruption, fetal death.

### 5.6 Search strategy

The search will be based on following keywords and Medical Subject Heading; “pregnant women”, “aspirin”, “aspirin placebo”, “Gestational hypertension”, “preeclampsia”, “eclampsia”, “HELLP syndrome”, “fetal growth restriction”, “placenta abruption”, “fetal death”. The following databases will be searched for eligible studies: Science direct, Medline, Embase, Pubmed, Africa Wide, Google scholar, ResearchGate, EBSCOhost, Web of Science, and the Cochrane Library, and LILACS. Medical subject headings (MeSH) and free text searches will be saved to the citation manager Zotero v5.0.81 (Zotero.org; Virginia). This software will be used to remove duplicates. The title and abstracts of the articles remaining after exclusion of duplicates will be assessed for eligibility according to the inclusion and exclusion criteria.

### 5.7 Study selection

The full text of all potentially eligible studies will then be reviewed by 2 independent reviewers (PZM, OPK), and any disagreement between reviewers with respect to eligible studies for inclusion in the analysis will be assessed for more eligible studies. Initially, studies will be screened by the titles, abstracts, keywords, and synonyms then followed by the identification of the full-text articles. Should discrepancies arise between 2 authors (PZM, OPK), a third author (WNP) will screen such studies, and consensus will be reached through discussion. Zotero v5.0.81 (Zotero.org) will be used to manage extracted data items, including saving relevant and excluded studies with reasons. Importantly, reference lists of included studies will be screened to confirm that no relevant studies are left out. Studies meeting the inclusion criteria will then be subjected to data collection, critical appraisal, risk, and quality evaluation.

### 5.8 Inclusion criteria

We will include relevant trials that are published in English regardless of publication status.

a. Cohort, case–control and randomized controlled trials with data on aspirin;
b. Studies with comparable aspirin and aspirin naïve populations;
c. Studies published in English and between 2004 to 2021;
d. Global studies involving participants aged >18 years;
e. Studies involving pregnant women on LDA only at gestations ≤14 and 36 weeks;
f. All of the criteria defining the impact of LDA only on HDP.

### 5.9 Exclusion criteria

a. Unpublished manuscripts and conference abstracts;
b. Studies whose data will not be sufficient to calculate appropriate measures of effect;
c. Studies which include LDA in combination with other drugs;
d. Evidence published before aspirin was introduced;
e. Cross-sectional studies;
f. Expert opinions and review/meta-analysis;
g. Where studies are suspected to have duplicate or multiple publication, only the most relevant; study pertaining to the objectives will be selected.

## 6 Data management

### 6.1 Data collection process

The reviewers (ZM and OPK) will develop a data extraction form that will be used in the collection relevant data items. To reduce data entry errors, selected studies will be independently assessed by 2 reviewers (ZM and OPK), the third reviewer (WNP) will be consulted for arbitration in case of any disagreements.

### 6.2 Data items

Extracted data items will include the author's name, year of publication, gestational age, parity, gravidity, body mass index, metabolites, commodity, supplements, socioeconomic status, compliance, maternal outcome, fetal outcome, and pharmacokinetics’ of aspirin.

### 6.3 Risk of bias in individual studies

To evaluate the potential risk of bias in randomized control trials, cohort, and matched cohort, Cochrane collaboration tool for assessing bias^[15]^ and Downs and Black checklist^[16]^ will be used. Two independent reviewers (PZM and OPK) will appraise all included studies and a third (WNP) reviewer will be consulted in cases of disagreements.

### 6.4 Data synthesis

A summary of findings table will be used to provide a synthesis of the main outcomes of included studies. Data will be analyzed with Rev Manager (Version 5.3; California) to conduct a meta-analysis. To measure statistical heterogeneity between studies, I^2^ and Chi squared statistical tests will be used.^[17,18]^ An I^2^ value of >50% will be considered substantial heterogeneity.^[19]^ To find the sources of heterogeneity within the included studies, a subgroup analysis and meta-regression comparing the study estimates from different study-level characteristics, quality, intervention type, and the reported effect measure of adverse events will be conducted.

## 7 Quality assessment of the cumulative evidence

The Grading of Recommendations, Assessment, Development and Evaluation assessment tool^[20]^ will be used to assess the overall quality of evidence. Moreover, the quality of each included study will be independently evaluated by 2 authors (PZM and OPK). The third author will adjudicate in cases of disagreements. The quality of evidence will be assessed based on several factors such as study limitations, indirectness of results, and publication or reporting bias. The evidence of each outcome will be rated as high, moderate, low, or very low.

## 8 Discussion

The prevalence of PE has been a major contributing factor in adverse pregnancy outcomes such as low birth weight, preterm birth, intrauterine growth restriction or small for gestational age, postpartum haemorrhage, stillbirth, and neonatal death.^[13]^ Therefore, the use of LDA as a prophylactic treatment has been considered for the prevention of PE. Administration of LDA should not be taken beyond 36 week to prevent possible adverse neonatal outcome. It is considered a low-dose when consumed at less than 300 mg/day.^[20]^ Low dose aspirin commenced at 16 weeks or earlier is associated with a significant decrease in relative risk for PE and effective in preventing early onset PE.^[21]^

Extensive work has been done involving different dosages of aspirin and combined with other drugs for prevention of PE.^[22,23]^ However, the effect of LDA alone on the prevention of PE in populations of African ancestry alone is currently limited. This review focuses on articles reporting on the effect of LDA in the prevention of PE without any drug combination. Moreover, the number of articles reporting aspirin compliance still remains limited and questionable on whether noncompliance may have an impact on LDA response. In addition, a limited number of studies reports on the effect of aspirin in pregnant women of African ancestry.

The proposed systematic review and meta-analysis will focus on randomized control trials, matched cohort, case control and observational studies that determine the effect of LDA initiated at 14 weeks of gestation. A similar literature review reported that the intake of LDA starting from or before 16 weeks of gestation is predominantly effective in preventing severe PE, requiring preterm delivery.^[24]^ However, this study had a small number of studies that fulfilled entry criteria and resulted in a limited meta-analysis. Most authors did not separately report preterm and term PE. Therefore this systematic review will focus on studies separately reporting both preterm and term PE.

## Author contributions

PZM, WNP, OPK, have outlined and prepared the draft of the protocol under the guidance of VD and supervision of JM. All authors contributed to the planning and design of the study. All authors edited and approved the protocol.

**Conceptualization:** Wendy Phoswa, Mkhize PZ.

**Formal analysis:** Wendy Phoswa, Mkhize PZ.

**Methodology:** Wendy Phoswa.

**Validation:** Wendy Phoswa.

**Writing – original draft:** Wendy Phoswa, Mkhize PZ, Khaliq OP.

**Writing – review & editing:** Wendy Phoswa, Mkhize PZ, Khaliq OP.
